# Partial excisional tapering: Report of initial four cases of a novel modification of ureteroplasty for megaureter

**DOI:** 10.1002/iju5.12658

**Published:** 2023-10-26

**Authors:** Motohiro Taguchi, Akihiro Kanematsu, Shingo Yamamoto

**Affiliations:** ^1^ Department of Urology Hyogo Medical University Hyogo Nishinomiya Japan

**Keywords:** megaureter, tapering, ureteroplasty

## Abstract

**Introduction:**

We report our initial experience with a novel ureteroplasty technique that combines the advantages of excisional tapering and folding.

**Methods and cases:**

Following dissection of the megaureter, the distal ureteral end was transected at a point with an appropriate caliber to create a neo‐orifice, which was left intact. Only the proximally redundant part of the ureter was excised and closed over a 10F catheter, following which the ureter was anastomosed to the bladder with an indwelling ureteral stent. This procedure was performed in four pediatric patients. Case 1 involved a 6‐year‐old girl with continuous urinary incontinence due to ureteral ectopia in a duplex system. Cases 2 and 3 involved infants with refluxing megaureter. Case 4 involved a 9‐year‐old boy with a ureteral stone impacted in a megaureter. All four patients achieved successful outcomes without ureteral obstruction.

**Conclusion:**

This ureteroplasty technique is a promising alternative in specific patients.

**Abbreviations & Acronyms**
CAP: continuous antibiotics prophylaxis
DJ: double J type stent
SJ: single J type stent
UCN: ureterocystoneostomy
UTI: urinary tract infection

**Keynote message**

This article describes a novel modification of ureteroplasty that combines the advantages of the two conventional methods of ureteroplasty: excisional tapering and non‐excisional folding.

## Introduction

UCN for megaureter repair is typically associated with ureteroplasty to facilitate the creation of a secure submucosal tunnel.^1^, ^2^ Excisional tapering and non‐excisional folding are two commonly used methods in ureteroplasty.^3^, ^4^, ^5^ Herein, we report a novel modification of ureteroplasty technique performed in four cases.

## Surgical method and case reports

**Fig. 1:**
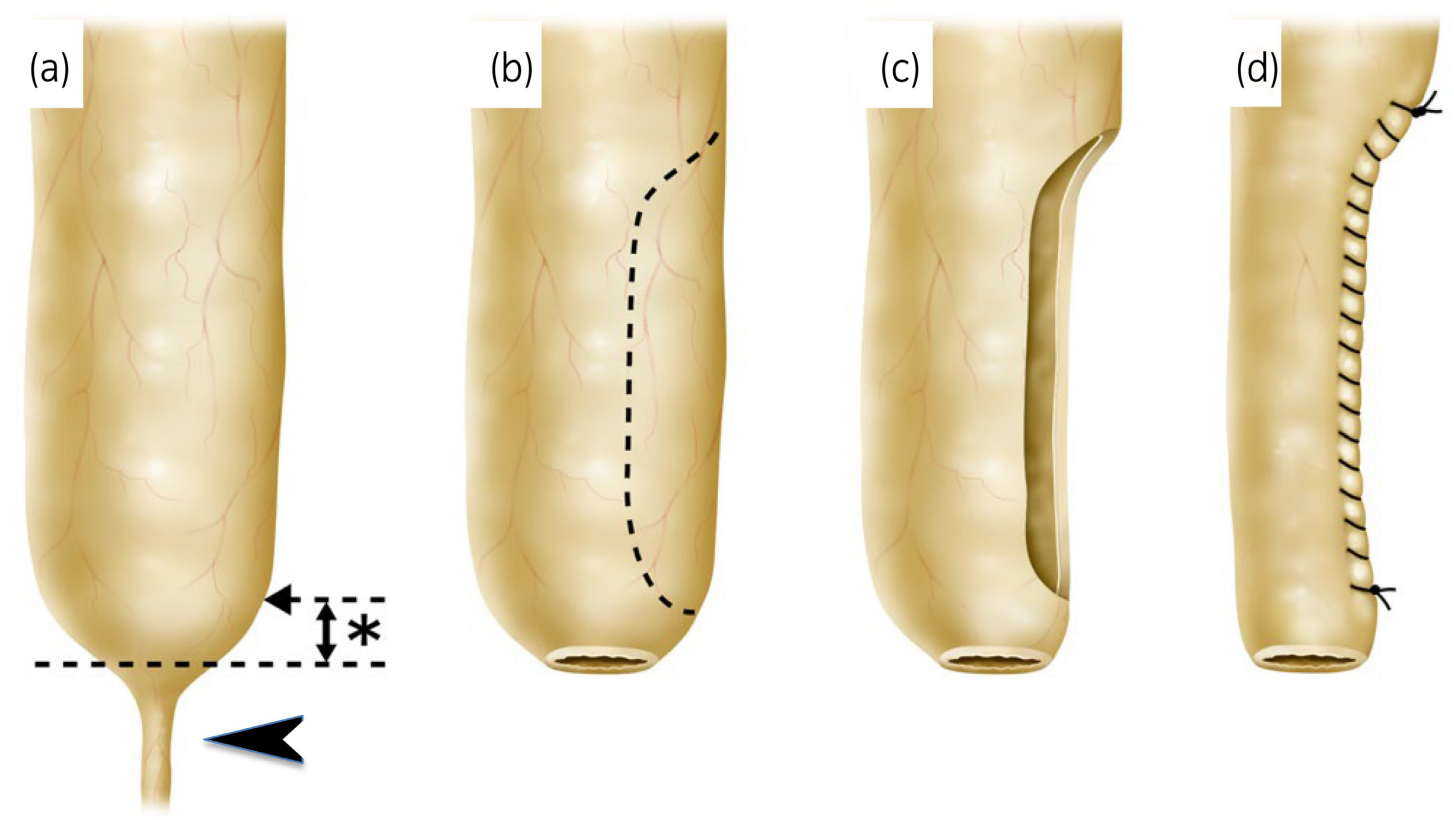
Schema of partial excisional tapering of a megaureter. (a) The stenotic part indicated by black arrow is completely discarded but the intermediate portion indicated by (*) is left intact, distal to the dashed arrow indicating the point of transection used in conventional methods (b). The ureteral end is transected at a level to create a neo‐orifice with a caliber to accommodate a 10Fr tube. (c) The ureter is trimmed only in the proximally redundant portion. (d) The defect is closed over a 10F catheter.

**Fig. 2:**
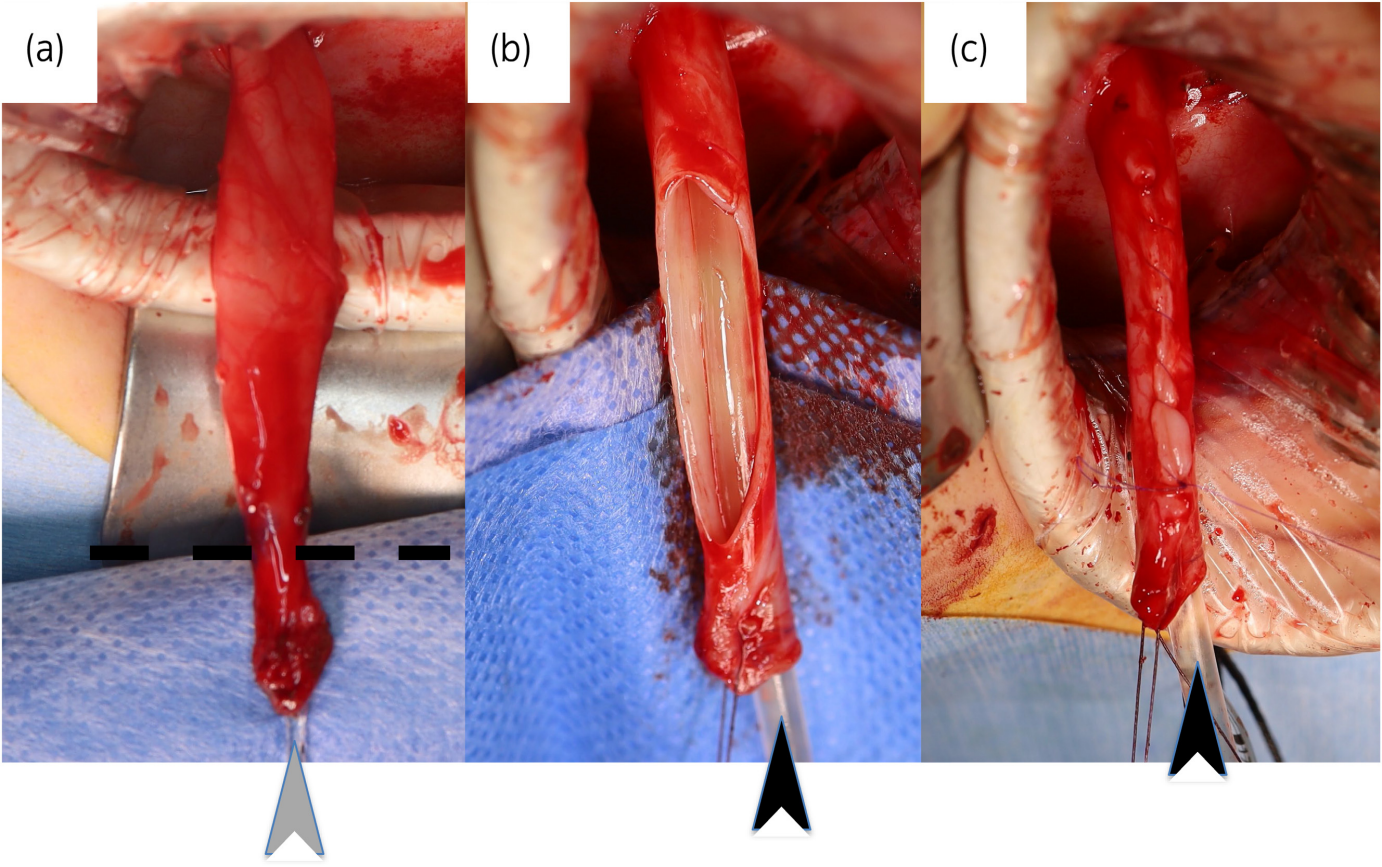
Case 2. Partial excisional tapering of the left ureter in a 1‐year‐old boy with refluxing megaureter. (a) After ureteral dissection with a 4Fr feeding tube (gray arrowhead) inserted in the lumen. Dashed line indicates the level of transection for creating neo‐orifice. (b) After discarding the distal stenotic portion, redundant ureteral tissue has been excised over a 10 F catheter (black arrowhead). (c) The ureter was tapered over the same 10 F catheter (black arrowhead).

Procedure (Figs 1 and 2): Following dissection of the megaureter, the distal ureteral end is transected at a level to create a neo‐orifice with a caliber to accommodate a 10Fr tube. Only the proximally redundant part of the ureter is excised and closed over the 10F catheter, after which it is anastomosed to the bladder with an indwelling ureteral stent. Four patients with different conditions underwent this procedure (Table 1).

**Table 1:** Summary of the cases

	Case 1	Case 2	Case 3	Case 4
Characteristics				
Sex	F	F	M	M
Age	6 y.o	10 m.o.	1 y 10 m.o.	10 y.o.
Presentation	Urinary incontinence	Febrile UTI	Febrile UTI	Microhematuria
Laterality	Left	Left	Left	Left
Diagnosis	Ectopic ureter (vagina) Duplex kidney	Refuxing megaureter with ectopia (bladder neck)	Refuxing megaureter	Megaureter Ureteral stone
Surgical Methods				
Approach	Intra and extravesical	Intravesical	Intravesical	Extravesical
Ureteral caliber (mm)	22	8	10	10
Tapering length (mm)	30	40	30	40
Suture material	7‐0 Maxon (Inner layer) 6‐0 Polyglactin (Outer layer)	6‐0 Polydeoxanone	6‐0 Polydeoxanone	5‐0 Poliglecapron
Suturing technique	Two‐layer running	Two‐layer running	Two‐layer running	One‐layer interrupted
Stenting catheter	4.7F DJ	6F SJ	6F SJ	4.7F DJ
Stenting period (weeks)	4	2	1	6
UCN	Cross‐trigonal	Cross‐trigonal	Cross‐trigonal	Extravesical
Postoperative course				
Follow‐up (m)	12	38	3	21
Pre/postoperative	0/0	1/1	0/0	0/0
Hydronephrosis grade				
Febrile UTI	None	Yes	None	Yes
Reoperation	None	None	None	Yes

Case 1. A 6‐year‐old girl presented with continuous urinary incontinence due to a duplex kidney with a vaginal ectopic opening of the upper‐pole ureter. Magnetic resonance imaging and contrast‐enhanced computed tomography revealed a duplex collecting system in the left kidney, with a massively dilated upper‐pole ureter. *En bloc* UCN of the two left ureters was performed. The lower‐pole ureter was dissected using an intravesical approach and then passed out to the extravesical space, where the dilated upper‐pole ureter was dissected near the vaginal opening. The dissected ureteral end had a several millimeter‐length portion with mild dilatation, which was left untouched. More proximally, the extremely dilated portion was tapered using partial excision. The two ureters were passed inside the bladder, and cross‐trigonal UCN was performed. The incontinence resolved immediately after surgery.

Case 2. Seven‐month‐old female infant presented with a recurrent febrile UTI. The patient had grade 1 hydronephrosis of the left kidney. A cystography confirmed the diagnosis of a refluxing megaureter opening in the bladder neck. The patient was maintained under CAP until the age of 10 months when UCN was performed. Following dissection of the left ureter, the ureter was narrowed using the partial excision technique, and a cross‐trigonal UCN was performed. After the surgical procedure, the patient remained free of CAP.

Case 3 (Fig. 2). Ten‐month‐old male infant presented with a recurrent febrile UTI. A cystogram demonstrated a refluxing megaureter. The patient was maintained under CAP until the age of 1 year 10 months when a cross‐trigonal UCN was performed for persistent reflux using the partial excision technique.

Case 4. A 9‐year‐old boy presented with microhematuria and was found to have a 10‐mm ureteral stone in the dilated lower ureter without hydronephrosis. Removal of the stone by a ureteroscopic procedure was planned, but retrograde imaging revealed that the stone was formed in the distal lumen of a megaureter, above a 10‐mm‐long narrow segment. Ureterolithotomy was performed in conjunction with megaureter repair. The left ureter was approached extravesically with a pararectus incision and secured using a vessel loop. The ureter was then incised longitudinally to remove the stone, and transection was performed at the non‐incised narrower portion. The opened ureteral mucosa was swollen owing to inflammation associated with stone impaction. The lower ureteral end was transected for the neo‐orifice, and the longitudinally opened ureteral wall was trimmed and closed over a 10F catheter with interrupted sutures. UCN was then performed extravesically. Six months postoperatively, the patient experienced febrile UTI due to *de novo* reflux. The patient underwent revision UCN with a psoas hitch, which did not require repeated ureteroplasty.

## Discussion

The present procedure combines the advantages of excisional tapering and folding methods. Although excisional tapering can create a thinner outer diameter of the ureter than folding, it has the potential risk of stenosis in the neo‐orifice due to ischemia by excision of the redundant ureteral tissue.^3^ A study that used a mini‐pig experimental model demonstrated that the folding technique is superior to excisional ureteroplasty in terms of better maintenance of blood supply.^6^ Another advantage of the folding method is the absence of a suture line at the neo‐orifice.

However, the folding of redundant ureter tissue sometimes results in a thicker outer caliber than excisional tapering, which may make it difficult to create a secure submucosal tunnel or anastomose the neo‐orifice to the bladder mucosa. The present procedure overcomes some of the drawbacks of these two methods, as the tissue continuity of the ureteral orifice is preserved, and redundant ureteral tissue is not undermined in the submucosal tunnel. Although it is not conclusive whether our method reduces the risk of ischemia in the neo‐orifice, the preservation of ureteral circumferential continuity at the neo‐orifice is likely to reduce the risk of unexpected stenosis following incision and suturing. This method preserves the intermediate ureteral portion between stenotic and dilatated portions, which is completely excised using conventional ureteroplasty techniques. Although the peristalsis is unknown for this intermediate part, it is wide enough to accommodate 10Fr tube, which is enough caliber for urine passage in tapering or folding method. Because the length of this portion varies among patients, attending surgeons must judge the applicability of this method based on the ureter shape. For example, it may not be suitable for dilated ureters associated with large ureteroceles, because they do not usually have such intermediate portions. Our initial experience with this method was successful in all four patients. The outcomes may not have been radically different from those of excisional tapering or folding because the procedure is based on the same principles known to produce satisfactory outcomes. Direct clinical comparisons between the two conventional methods are scarce, and the attending surgeon typically employs either or both methods based on personal preferences and experience. Thus, the advantages and disadvantages of both methods are largely speculative, similar to the advantages of the present technique. With this in mind, we propose that this novel modification should be considered as an alternative option, particularly in difficult cases with highly redundant ureteral tissue, such as in Case 1, or severely swollen mucosa, such as in Case 4, although it can also be applied for ordinary refluxing megaureter cases, such as in Case 2 and 3. Further accumulation of cases will establish the usefulness of this procedure in pediatric urology practice as a surgical alternative of ureteroplasty.

## Author contributions

Motohiro Taguchi: Data curation; visualization; writing – original draft. Akihiro Kanematsu: Conceptualization; data curation; methodology; project administration; supervision; writing – original draft; writing – review and editing. Shingo Yamamoto: Project administration; writing – review and editing.

## Conflict of interest

The authors declare no conflict of interest.

## Approval of the research protocol by an Institutional Reviewer Board

This study was approved by the Ethics Committee of the Hyogo Medical University (approval number 20103‐040).

## Informed consent

Informed consent from the patients and guardians was obtained using an opt‐out method.

## Registry and the Registration No. of the study/trial

Not applicable.
